# Role of time-dependent foreign molecules bonding in the degradation mechanism of polymer field-effect transistors in ambient conditions

**DOI:** 10.1098/rsos.221272

**Published:** 2023-06-14

**Authors:** Muhammad Zohaib, Tahmina Afzal, M. Zahir Iqbal, Badriah S. Almutairi, Mohsin Ali Raza, Muhammad Faheem Maqsood, M. Akram Raza, Saira Riaz, Shahzad Naseem, M. Javaid Iqbal

**Affiliations:** ^1^ Centre of Excellence in Solid State Physics, University of the Punjab, Quaid-e-Azam Campus, Lahore 54590, Pakistan; ^2^ Nanotechnology Research Laboratory, Faculty of Engineering Sciences, GIK Institute of Engineering Sciences and Technology, Topi-23640, Khyber Pakhtunkhwa, Pakistan; ^3^ Department of Physics, College of Science, Princess Nourah bint Abdulrahman University, P.O. Box 84428, Riyadh 11671, Saudi Arabia; ^4^ Department of Metallurgy and Materials Engineering, University of the Punjab, Lahore-54590, Pakistan

**Keywords:** organic field-effect transistors, contact resistance, channel resistance, FTIR, polymer conjugation perturbation, ambient instability

## Abstract

Long-standing research efforts have enabled the widespread introduction of organic field-effect transistors (OFETs) in next-generation technologies. Concurrently, environmental and operational stability is the major bottleneck in commercializing OFETs. The underpinning mechanism behind these instabilities is still elusive. Here we demonstrate the effect of ambient air on the performance of p-type polymer field-effect transistors. After exposure to ambient air, the device showed significant variations in performance parameters for around 30 days, and then relatively stable behaviour was observed. Two competing mechanisms influencing environmental stability are the diffusion of moisture and oxygen in the metal–organic interface and the active organic layer of the OFET. We measured the time-dependent contact and channel resistances to probe which mechanism is dominant. We found that the dominant role in the degradation of the device stability is the channel resistance rather than the contact resistance. Through time-dependent Fourier transform infrared (FTIR) analysis, we systematically prove that moisture and oxygen cause performance variation in OFETs. FTIR spectra revealed that water and oxygen interact with the polymer chain and perturb its conjugation, thus resulting in degraded performance of the device upon prolonged exposure to ambient air. Our results are important in addressing the environmental instability of organic devices.

## 1. Introduction

The inherent properties of organic semiconductors (OSCs), such as mechanical flexibility, low-temperature processability, lightweight and tuneable band gap, have attracted the enormous attention of researchers to the emerging technology of organic electronics [1–7]. The organic field-effect transistors (OFETs) are the backbone of the devices based on OSCs. The potential applications of OFETs are active-matrix backpanels, flexible displays, liquid crystal displays, solar cells, light-emitting diodes [8,9], logic circuits for the applications such as radio frequency identification tags [10], and chemical and biological sensors [11].

Despite the vast potential applications, the OFETs suffer from two major limitations, low-charge carrier mobility and environmental and operational instabilities. The charge carrier mobility is significantly enhanced by the use of a blended polymer in the active channel layer [12], incorporation of ionic additives/graphene interlayer [13,14], optimizing the annealing temperature of polymers [15], using trap-free dielectric layer [16], and/or by doping the contact areas with the metal oxide layer/self-assembled monolayer during the fabrication of OFETs [17]. The charge carrier mobility of OFETs has exceeded that of amorphous silicon-based devices with values greater than 10 cm^2^ (V.s)^−1^, making them competitors of the inorganic or metal oxide-based field-effect transistors (FETs) [18]. However, long-term environmental and operational instability hindered the widespread commercial deployment of OFETs. These instabilities cause a shift in threshold voltage, an increase of the OFF-state current, suppression of field-effect mobility, and/or a decrease in the sub-threshold swing. These instabilities mainly originate from the adsorption of atmospheric species such as oxygen or water molecules in the organic layer, the presence of oxygen/water molecules onto the surface of the dielectric, sensitivity to light exposure, variations in the metal–semiconductor contact areas, or a combination thereof [19,20]. Nevertheless, there are gaps in the literature to provide clear evidence for the cause of these instabilities. In this study, we employed Fourier transform infrared (FTIR) analysis to prove the cause of environmental instabilities systematically. We have correlated the effect of environmental oxygen and water adsorption to the changes in the contact resistance and the channel resistance. As a result, the change in the resistance values changes the performance parameters of the OFETs.

The trapping of charge carriers owing to the presence of atmospheric species in the OSC and organic–dielectric interface has been substantially researched, and its effect on the performance of OFET devices has been widely discussed [21,22]. However, the effect of these atmospheric species on the metal–semiconductor contacts remained elusive. Significant attention is needed to explore the variability of contact resistance upon prolonged exposure to ambient air. Nikolka *et al*. showed that the contact resistance of top-gate bottom-contact OFET decreased upon exposure to ambient air for 24 h [23]. Yu *et al*. investigated the contact resistance of bottom-contact pentacene-based FET for 30 days and showed that the performance degradation of the device is mainly owing to the increase in contact resistance. The increased surface roughness of pentacene film on top of source/drain electrodes caused the poor injection of charge carriers upon exposure to ambient air [24]. Mai *et al*. used the C8-BTBT (2,7-diocty[1]benzothieno[3,2-b][1]benzothiophene)-based bottom-gate top-contact FET to investigate the effect of different ambient gases on the performance of the devices. They showed that the performance variation is mainly owing to water molecules in the air. The contact resistance decreased upon exposure to ambient air for 2 h, and as a result, mobility increased. However, upon further exposure, they observed a decrease in carrier mobility which was attributed to the degradation of the channel by moisture [25]. These controversies in the literature regarding the effect of air exposure on the performance of OFET must be addressed appropriately.

Here we investigated the effect of ambient air on the contact and channel resistance of OFET and correlated the impact of these resistances on the variability of performance parameters of the device. We also probed the influence of moisture and oxygen onto the active organic layer of OFET as a function of storage time. With the help of FTIR analysis over time, we found that oxygen and water molecules from the ambient air bond with the polymer chains that as a result changes contact and channel resistance values. The detailed analysis of contact and channel resistance and their effect on the performance parameters of the device showed a correlation. In this report, we demonstrate for the first time to our knowledge, how O_2_/H_2_O molecules from air cause instability in the performance of OFETs proved by FTIR measurements. By exploiting the correlation between performance parameters and resistance, we unravel the contributions and mechanisms at different parts of OFETs.

## 2. Results and discussion

**Figure 1.**
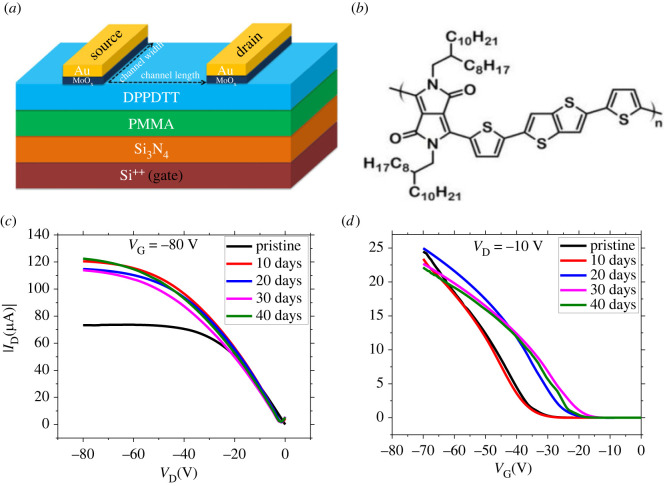
Performance evaluation of OFET under the ambient environment showing (*a*) schematic illustration of the fabricated device with the polymer DPP-DTT acting as a semiconducting channel layer. The channel length *L* and channel width *W* are 120 µm and 3 mm, respectively. (*b*) Chemical structure of DPP-DTT. (*c,d*) Output characteristics (*V*_GS_ = −80 V) and transfer characteristics in the linear regime (*V*_DS_ = −10 V) of the device obtained after the continuous characterization for 40 days at an interval of 10 days.

To investigate the effect of ambient air on the performance of OFETs, the poly[25-(2-octyldodecyl)-36-diketopyrrolopyrrolealt-55-(25-di(thien-2-yl)thieno 32-bthiophene) (DPP-DTT) was employed as an organic material. Poly(methyl methacrylate) (PMMA) was used as a second dielectric layer to improve the dielectric properties and suppress the interfacial trapping [16,26]. The PMMA layer was deposited onto the highly doped silicon substrate with pre-grown 400 nm-thick silicon nitride (first dielectric layer). The DPP-DTT polymer was then spin coated on the PMMA layer, followed by post-deposition thermal annealing at 100°C. To fabricate the bottom-gate, top-contact transistors based on the DPP-DTT polymer, the molybdenum oxide (MoO_3_) and gold (Au) were thermally evaporated onto the film through a shadow mask (see Material and Methods). Figure 1*a* illustrates the schematic of the fabricated device. The molecular structure of DPP-DTT is shown in figure 1*b*.

The freshly prepared OFET device was characterized to get the transfer and output characteristic curves. The device showed a strong p-type behaviour. The threshold voltage (*V*_th_) was determined from the x-intercept of the transfer curve's linear fit. The field-effect mobility was extracted from the transfer curve in the linear regime (│*V*_DS_│<│*V*_G_-*V*_th_│) using the gradual channel approximation model:

2.1 $$\mu_{\mathrm{lin}}=\frac{L}{C_{\mathrm{in}}V_{d}W}\frac{\partial I_{D,\mathrm{lin}}}{\partial V_{G}},$$

where *μ*_lin_ and *C*_in_ are the charge carrier mobility in the linear regime and capacitance per unit area of dielectric, respectively. The factor $\partial I_{D,\mathrm{lin}}/\partial V_{G}$ is the slope of the transfer curve (*I_D_*_,lin_ versus*V_G_* in linear scale). The effective mobility was calculated by the method described in [27].

Next, we let the OFET device age in the ambient environment (humidity 30–40%, temperature 24–28°C) for 40 days and kept characterizing the device at an interval of 10 days during this ageing period. Figure 1*c* shows the combined output characteristic curves at −80 V gate voltage, where pristine is referred to the immediate characterization of the device after fabrication, and the plots after the 10, 20, 30 and 40 days are also shown. The pristine device was operated in the linear regime, the channel was pinched off at around −40 V and then the saturation regime occurred. After the exposure to the ambient environment, the saturation regime was shifted to more than −55 V drain voltage, as shown in figure 1*c*. From the figure, it can be clearly observed that there are large fluctuations in the output curves of the aged device compared to the pristine device. Similarly, figure 1*d* shows the combined transfer curves in the linear regime. We observed significant variation in the performance parameters over time. The fluctuations in the transfer and output curves after prolonged exposure to the ambient environment are mainly attributed to the adsorption of oxygen and water molecules from the air [27], which cause the change in contact resistance by unintentional doping at contact areas, change in carrier concentration within the channel layer and/or adsorption of oxygen/water molecules through the dielectric layer [22]. We employed PMMA, which lowers the interfacial traps, and the architect of our device is the bottom gate, and top contacts, so the induction of oxygen/water molecules through the dielectric layer is significantly reduced [27]. We studied contact resistance and channel resistance to understand whether these fluctuations are owing to the diffusion of moisture or oxygen molecules in the contact areas or diffusion in the channel layer. We correlated the results with the fluctuations observed in the performance of OFET with time.

**Figure 2.**
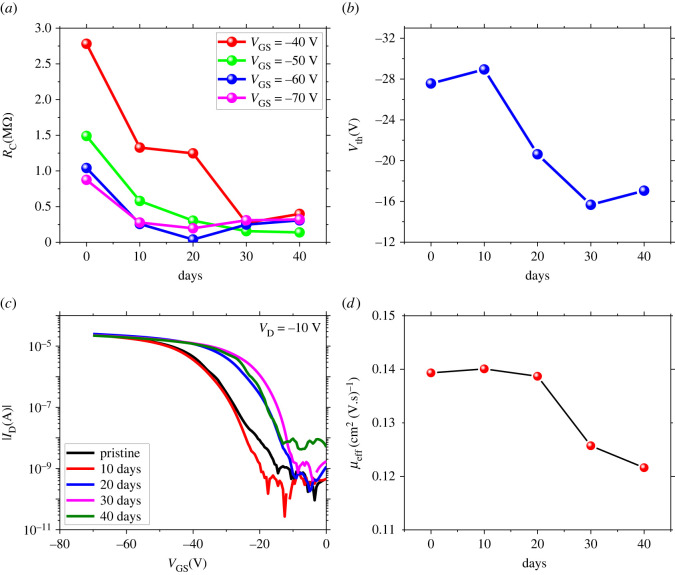
The comparison of performance parameters of OFET with contact resistance (*R*_C_) to check the influence of ambient environment on *R*_C_ and the effect of *R*_C_ on other parameters. (*a*) Contact resistance at different gate voltages as a function of time calculated by the TVM method. Here 0 days corresponds to the as-fabricated device. The contact resistance gradually decreases for the first few weeks and then saturated when the device is further exposed to ambient environment. (*b*) Threshold voltage as a function of time. It first decreases for the first couple of weeks and at the end saturates showing a similar trend as that observed for *R*_C_. (*c*) On-/off-current ratio shows a gradual decrease when device is preserved in ambient air. (*d*) Effective mobility calculated in linear regime showing a decrease trend in the first few weeks and then almost saturated (see the electronic supplementary material, table S1 for more information).

Different methods are used to calculate contact resistance, such as the transfer line, gated four probe, differential and transition voltage. For a detailed understanding and limitations of these methods, refer to the review by Natali & Caironi [28]. We used the transition-voltage method (TVM) to estimate the contact resistance proposed by Wang *et al*. [29]. The ease of using this method is that it does not require the intricate patterning of channel dimensions needed for the other methods, for example, the transfer-line method. It employs output and transfer characteristic curves for the calculation of contact resistance using:

2.2 $$R_{C}=\frac{2\sqrt{V_{G}-V_{T}}\left(\sqrt{2V_{\mathrm{tr}}}-\sqrt{V_{G}-V_{T}}\right)}{I_{D,\mathrm{sat}}},$$

where *R*_C_ is the contact resistance, which is the sum of the source (*R*_S_) and drain resistance (*R*_D_), *V*_G_, *V*_T_ and *V*_tr_ are the gate, threshold and transition voltages, and *I*_D__,sat_ is the saturated drain current. This method is shown to be as reliable as the transmission line method in literature when applied with certain limits [29–31]. Basic approximations of this method and contact resistance extraction by this method for our devices are discussed in the electronic supplementary material. Figure 2*a* shows the contact resistance (*R*_C_) as a function of exposure time (days) to ambient environment at different gate voltages, where 0 days represents the pristine device. We observed a drastic reduction in contact resistance during the first few weeks of exposure to ambient air, and then it gets saturated for all the gate voltages upon further exposure to ambient air. This reduction in *R*_C_ is attributed to the induction of oxygen/water molecules in the contact areas, which act as p-type dopants giving rise to better charge carriers injection [32]. It is usually observed that the thermal deposition of metal contacts onto the OSCs may produce damage and create trap states within the metal–semiconductor junction [33]. From our results, we may attribute the reduction in *R*_C_ to the diffusion of oxygen/water molecules in these trap states created by thermal damage. It is also reported that the work function of molybdenum oxide (MoO_x_) reduced from 6.8 eV to 5.6 eV upon 1 h of air exposure [34]. This reduction of 1.2 eV in work function was credited to the adsorption of moisture and oxygen from the air into the MoO_x_ thin film. This drastic reduction in work function lowered the energy levels mismatch between the electrode and semiconductor, which caused the better injection of carriers and reduced the contact resistance. We observed a decrease in the density of trap states which indicates a positive effect of the adsorption of O_2_/H_2_O molecules at the metal–polymer contact interfaces (electronic supplementary material, figure S3(b)). As the traps fill with time, it becomes easier for the injected charge carriers to reach the accumulation layer giving rise to a decrease in *R*_C_ for the first few weeks. When almost all the traps are filled, a saturation point occurred where no more reduction in *R*_C_ is observed after further exposure to ambient air, as depicted in figure 2*a*. From the results, we conclude that the reduction in contact resistance is attributed to the filling of thermally damaged trap states at the metal–polymer interface [32] and/or the work function modification of metal contacts [34] owing to the adsorption of moisture/oxygen molecules. However, the degree to which one of these mechanisms dominantes is non-trivial to conclude here and needs an extensive research to understand it explicitly.

Another significant trend we observed was the interesting behaviour of gate-dependent contact resistance. The *R*_C_ gradually decreases with increasing gate voltage for the first couple of weeks, which might be attributed to two factors. First, the increase in channel conduction causes the injection of carriers via the standard current crowding formalism [35,36]. Second, the increase in *V*_G_ inject more carriers in the semiconductor layer, thus high-carrier concentration within the metal–semiconductor junction causes the thermionic to field emission (tunnelling) injection of carrriers which causes the decrease in *R*_C_ [36,37]. However, when the OFET device was left to age for almost 30 days, no gate dependence of the contact resistance was observed, as shown in figure 2*a*. These results also strengthen the argument that oxygen/water molecules fill the active trap states at the metal–organic interface. Thus, the gate voltage dependence of *R*_C_ is present for the first couple of weeks. Yet, it is clear from the data that almost all the trap states at the metal–organic interface have been filled after 30 days of storage by unintentional contact doping of water–oxygen molecules, as can be concluded from the *V*_G_ invariant contact resistance (figure 2*a*).

To gain insight into the effect of contact resistance on the performance of OFET, we examined the performance parameters of OFET, such as threshold voltage, on-/off-current ratio, sub-threshold swing and charge carrier mobility. Figure 2*b* shows the threshold voltage (*V*_th_) as a function of exposure time. For the pristine device, many trap states are available within the OSC channel layer close to the dielectric interface. Initially, when the trap states are very abundant, a significant portion of gate voltage (*V*_G_) is consumed to fill these trap states, thus resulting in a large value of threshold voltage (*V*_th_ = −34 V). Up to 10 days of air exposure, no significant change in *V*_th_ was observed. However, as the device is further exposed to ambient air, these trap states are filled by moisture and/or oxygen molecules. Thus, a significant portion of gate voltage (*V*_G_), which was initially consumed to fill these trap states, is no longer required [20,37,38]. As a result, the device turns on with less gate voltage and threshold voltage reduced to approximately− 20 V and then is almost saturated over further exposure to air.

Brown and co-workers have developed a model for the on-/off-current ratio, in which they discussed two limiting cases depending on the doping level of the semiconductor [39]. For the case of low doping level (as in our case), the on/off ratio is given by

2.3 $$\frac{I_{\mathrm{on}}}{I_{\mathrm{off}}}=\frac{\mu }{\sigma }\frac{C_{\mathrm{in}}^{2}V_{d}^{2}}{qN_{a}t^{2}},$$

where *σ* is the conductivity, *q* is the elementary charge, *N*_a_ is the acceptor density and *t* is the thickness of the semiconducting layer. It can be seen that the on/off ratio not only depends on the ratio of mobility to conductivity, but other factors such as semiconductor thickness, dielectric capacitance and acceptor density also influence it. As in our case, the thickness, capacitance and ratio of mobility to conductivity were the same. The acceptor density is the main contribution to the on-/off-current ratio. Figure 2*c* provides the graph between gate voltage and drain current on a logarithmic scale. No significant change in on-current was observed, but the off-current was increased by approximately 10^2^ when exposed to ambient air for 40 days. Based on the FTIR data (discussed later), we believe that this reduction in on-/off-current ratio is owing to the increase in acceptor density, which provides evidence of diffusion of oxygen–water molecules into the semiconductor region of OFET [40]. We also calculated the sub-threshold swing of our device to account for the switching speed as a function of exposure time (electronic supplementary material, figure S3(a)). We observed a decrease in sub-threshold swing, indicating a change in trap states' density. The suppression in the density of states (electronic supplementary material, figure S3(b)) gives a clue about the filling of traps as a function of air exposure. Our results are consistent with the literature [32]. This is also consistent with the contact resistance that also reduces as the traps are filled.

One of the key parameters for assessing the performance of OFET is its charge carrier mobility. The nonlinearity in the transfer curves of the OFETs causes the inaccurate and unreliable extraction of performance parameters, especially charge carrier mobility. In order to avoid these issues, we used a method to calculate the effective mobility through the reliability factor from the transfer curves of OFET [27]. Figure 2*d* shows the trend of effective mobility versus exposure time extracted using the above method. The pristine device shows a mobility value of 0.14 cm^2^ (V.s)^−1^, reducing to 0.125 cm^2^ (V.s)^−1^ after 30 days of exposure and then is almost saturated. The injection is improving as the contact resistance decreases with the exposure time, so it should also increase mobility. However, the results are somehow opposite, *μ* is decreasing. From the results, we can conclude that the effect of contact resistance is negligible on mobility degradation [41]. If the contact resistance does not cause the degradation in mobility, then which factors are involved? To address this question, we extracted the channel resistance of the device.

**Figure 3.**
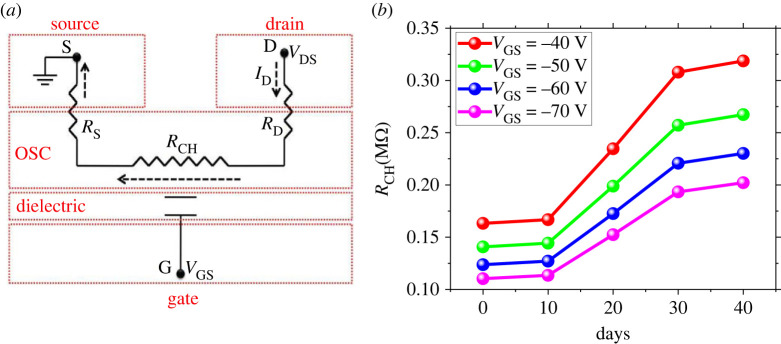
Analysis of channel resistance. (*a*) Equivalent circuit model where red dotted blocks correspond to the schematic of OFET shown in figure 1*a*. Three terminals for biasing are labelled as S, D and G. The contact resistance (*R*_C_) is the sum of source (*R*_S_) and drain resistance (*R*_D_). Dotted arrows show the direction of drain current. Note that *R*_C_ (present at the metal–semiconductor interface) is not the only opposition for the flow of current; another opposition also exists in the bulk semiconductor channel called the channel resistance *R*_CH_. (*b*) The channel resistance as a function of time at different gate voltages is shown.

The equivalent circuit model of the OFET shown in figure 3*a* depicts that two resistances limit the charge carriers’ transport from the drain to the source terminal through the OSC. One is the contact resistance (sum of *R*_S_ and *R*_D_) present at the contact areas, and the other is the channel resistance (*R*_CH_) in the semiconductor channel layer. Mobility is the ease of carrier transport under the applied electric field. Although the contact resistance decreases with time and results in the ease of carrier transport through contact interfaces, the charge carrier mobility is still decreasing. It means that mobility is not limited by metal–semiconductor contact areas. It is significantly influenced by the semiconductor channel layer. To prove this argument, we extracted the channel resistance by the G-function method [42]. For comparison with contact resistance, channel resistance as a function of exposure time is extracted in the linear regime of OFET operation at different gate voltages, as shown in figure 3*b*. After immediate characterization of the fabricated device, the *R*_CH_ was observed to be 0.16 MΩ at *V*_GS_ = −40 V. After the exposure to ambient air for about 30 days, *R*_CH_ increased to 0.3 MΩ and then almost saturated upon further exposure. A similar but opposite trend was observed for contact resistance behaviour with time at different gate voltages. As the OFET device we employed has a channel length *L* > 10 µm, the significant role of mobility degradation is channel resistance rather than contact resistance [43,44].

The increase in channel resistance might be attributed to the moisture and/or oxygen diffusion within the organic channel layer. It is widely considered that the leading cause of the performance variations of OFET in the ambient environment is the adsorption of water/O_2_ molecules in the organic layer [45,46]. However, there is no evidence-based study on how exactly the foreign molecules attach to the polymers and cause performance degradation in OFETs. Here we employed FTIR spectroscopy to investigate how water/O_2_ molecules bond with the organic layer of the device, thus limiting charge transport.

**Figure 4.**
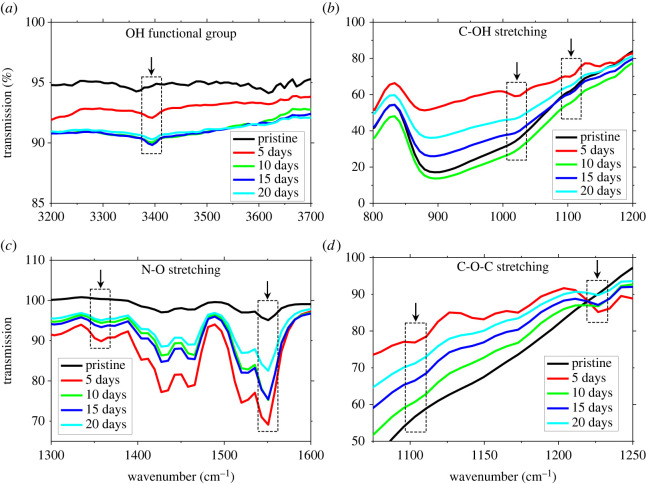
FTIR analysis of PMMA/DPP-DTT film recorded as a function of storage time in an ambient environment. (*a*) The arrow shows the peak around wavenumber 3400 cm^−1^ corresponds to the OH-bond evident in the adsorption of water molecules in the film. The peak gets intense and broadens with the passage of time. (*b*) The arrows around 1026 cm^−1^ and 1103 cm^−1^ show the stretching of the C-OH bond and describe the interaction of water molecules with polymer material; (*c,d*) for nitro and ethers, respectively.

In order to investigate the presence of water and oxygen molecules into the organic channel layer, the FTIR analysis was performed as a function of ageing time of the device. The PMMA/DPP-DTT were deposited onto the glass substrate and FTIR spectra were obtained in the attenuated total reflection (ATR) mode for 20 days, as depicted in figure 4. The FTIR spectra between the transmission and wavenumber give the unique fingerprint for polymer materials' chemical structure [47]. The 3400 cm^−1^ wavenumber corresponds to the OH functional group. Figure 4*a* shows a small OH peak for pristine film around 3400 cm^−1^, as the film is stored in ambient air, the OH peak becomes more intense and broadened with the passage of storage time. These results showed the penetration of moisture into the polymer layer. Figure 4*b* shows the interaction of adsorbed moisture with a polymer layer. The arrows show the band stretching for the C-OH bond around 1026 cm^−1^ and 1103 cm^−1^ wavenumber. No significant band stretching is observed for pristine film, but as the storage time increases, band stretching appears, and then it broadens and saturates for prolonged exposure to ambient air (figure 4*b*).

Another significant interaction of moisture and oxygen with the polymer layer was observed in the form of oxygen. From the structural formula of DPP-DTT as shown in figure 1*a*, it can be seen that nitrogen is only bonded with carbon. However, we found sign of nitro compounds in the infrared spectrum of the aged device. Figure 4*c* shows the FTIR spectra of the sample as a function of storage time. There is no band stretching around 1357 cm^−1^ for the pristine device. As the device is aged for 5 days, an intense peak appears at 1357 cm^−1,^ which finally saturates in prolonged storage to ambient air. This peak signifies adsorption and oxygen interaction with nitrogen atoms to form symmetric nitro compounds. Another interaction of oxygen with nitrogen is observed around 1550 cm^−1^, which corresponds to the asymmetrical stretching of bands for nitro compounds [48]. The peak is very small at 1550 cm^−1^ for the pristine device. We suspect that because all the fabrication process was done in ambient air, this peak may have come from oxygen interaction with nitrogen before the characterization. As the device is aged in ambient air, this peak intensifies with storage time, as shown in figure 4*c*. Two peaks at 1357 cm^−1^ and 1550 cm^−1^ are evidence of the interaction of oxygen with an important constituent (nitrogen) of the polymer chain.

The chemisorption of oxygen is not limited to the formation of nitro compounds but also extends to the formation of ethers. The FTIR region from 1000 to 1300 cm^−1^ corresponds to the ethers and alcohols [49]. Figure 4*d* shows the infrared spectra as a function of storage time. There is no peak observed for the pristine device around 1103 cm^−1^ and 1226 cm^−1^. As the device is aged for 5 days, two intense peaks appeared at 1103 cm^−1^ and 1226 cm^−1^, corresponding to C-O stretching for saturated and aryl ethers, respectively [50]. Note the trend of these peaks, which are comparatively intense for early storage and broadens for the aged device. From the results, we expect that the formation of ethers owing to the chemisorption of oxygen may have changed the conjugation of the polymer layer. The change is large for the first few days and then comparatively small change correlates with the large degradation in the device performance for the first few weeks, and then almost no change is observed for prolonged exposure of the device to ambient air. The variations in the intensity of the FTIR spectra could be attributed to the fact that the humidity levels will be somewhat different and also a different area of the organic film is scanned each time FTIR spectra is taken, which will take into account the slight variation in the composition of the film. It is widely reported that small organic molecules such as pentacene and rubrene undergo oxidation when exposed to ambient air [51,52]. This oxidation significantly degrades the device performance[53,54]. Here we focused on the conjugated polymer DPP-DTT and found that the chemisorption of oxygen from the environment results in forming ether and nitro compounds. The creation of these compounds has perturbed the conjugation of the polymer and hence impedes charge transport.

From the results, we can conclude that the interaction of oxygen with the polymer layer upon prolonged exposure to ambient air may have partially broken the chains, affecting the polymer layer's conjugation. These results strengthen the argument that the initial degradation in the performance parameters of the OFET device is owing to the adsorption of moisture and oxygen in the polymer layer. These FTIR results correlate nicely to performance parameters**'** behaviour, as after exposure to ambient air, the large variations in performance parameters are relative to adsorption and moisture interaction with the organic channel layer. However, for extended exposure time, no significant variations in performance parameters of OFET indicate the saturation of moisture interaction with the organic channel layer, as shown in figure 4*a,b*.

**Figure 5.**
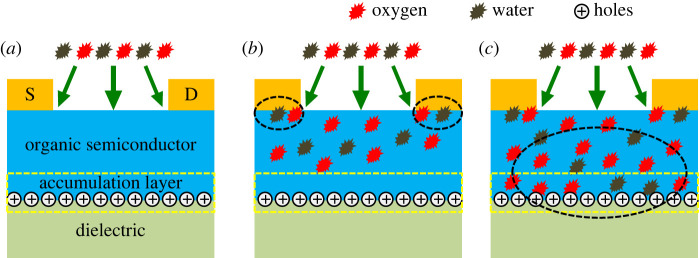
Schematic illustration of moisture and oxygen diffusion into the exposed area of the OFET device. (*a*) Labelling of the device showing the source and drain electrodes where the plus signs denote the channel formed by p-type charge carriers. The region between the dotted yellow lines shows the accumulation layer at the semiconductor–dielectric interface. (*b*) Initial diffusion of moisture/oxygen into the metal–organic contact interface shown by black circles and into the upper part of organic semiconducting layer around two weeks of air exposure. (*c*) After continuous exposure of more than two weeks, the moisture/oxygen molecules penetrate deep into the channel layer.

Figure 5 illustrates the interaction of moisture and oxygen with OFET. Figure 5*a* shows the schematic labelling of the layers for the pristine device. When the device is stored for about two weeks in ambient air, the moisture/oxygen is adsorbed on the polymer's upper layer. Initially, the moisture/oxygen interacts with contact areas shown by dotted circles. Owing to this interaction, an abrupt change in contact resistance was observed for about two weeks as shown in figure 2*a*. Then no more change was observed in *R*_C_ when the device was further exposed to air. Similarly, when the moisture/oxygen has not yet reached the accumulation layer for about two weeks, there is no significant variation in performance parameters and channel resistance at this stage. Also, the density of trap states reduced significantly during the initial exposure owing to the filling of traps at the metal–polymer interface (electronic supplementary material, figure S3(b)), which kept an insignificant change in the performance of OFET dominated by improvement of contact areas. However, when the moisture/oxygen reached the accumulation layer after more than two weeks of exposure to ambient air, as shown in figure 5*c*, an abrupt variation in performance and channel resistance was observed, dominated by the disruption in the conjugation of polymer chains. Overall, although a positive contribution of O_2_/H_2_O at the contact interfaces tried to improve the performance of OFET, the negative contribution of these foreign molecules within the polymer channel dominated the degradation of our device. After further exposure, no more variation indicates that the device's performance has reached saturation point.

### 2.1. Summary

To close our discussions in a nutshell, we summarize the results as follows:

(i) the solution-processed OFETs fabricated and stored in ambient air for a prolonged time showed notable performance variations;
(ii) the moisture-induced performance variations may arise owing to moisture and/or oxygen interaction at two areas of the bottom-gate, top-contact OFET device. One is at the metal–organic interface area shown in figure 5*b* by dotted circles. The second is at the organic semiconducting channel layer shown in figure 5*c* by a dotted circle;
(iii) after investigating the contact resistance behaviour with storage time, we found that moisture positively reduces contact resistance by acting as a dopant and/or modifying the MoO_x_ work function. These dopant molecules filled the trap states at the metal–organic interface, which facilitated the injected charge carriers to reach the accumulation layer, resulting in the gate voltage-independent contact resistance for prolonged storage time. The positive effect of moisture/oxygen in reducing the *R*_C_ and obtaining the *V*_G_ invariant *R*_C_ will open up a new path for achieving a device with better contacts and ideal *V*_G_-dependent behaviour for the OFETs; and
(iv) finally, we performed FTIR spectroscopy to verify the presence of moisture and oxygen in the organic channel layer. FTIR analysis provided evidence of adsorption and interaction of the oxygen and water molecules with the polymer material. We anticipate that this interaction between the moisture/oxygen and polymer may have changed the polymer film's conjugation; thus, degradation in performance parameters was observed. However, this needs a better scientific understanding of what extent both water and oxygen contribute to the performance variations and how both species will behave for different organic materials.

## 3. Conclusion

Ambient instability in OFETs has puzzled researchers for the last two decades. The difficulty in achieving stability lies in understanding the factors that cause degradation in the performance of the devices. Although the origin of degradation has been suggested to be the interaction of the polymer with the water molecules in ambient air, there is little experimental evidence available on how the external molecules attach to the conducting polymers and degrade their performance. Herein, we show, with the help of FTIR spectra, that the diffusion of water molecules disrupts the conjugation of the polymer by introducing different groups which were not present in the fresh polymer film. However, their presence was verified when the devices were stored and operated in the ambient environment. Further, we showed that channel resistance plays a dominant role in degrading the device performance rather than contact resistance in long-channel devices. Thus, our findings are important to understand the primary mechanism of device degradation. This report pinned the mechanisms occurring at the metal–polymer interface and within the polymer chain to quantify the variability in the performance of polymer FETs when stored in the ambient environment. Although we have provided a way to achieve stability in OFETs by ageing them, the initial degradation can also be addressed by pointing out the actual cause of degradation. Hence, this study will be a significant step forward in understanding and achieving ambient stability in OFETs.

## 4. Material and methods

### 4.1. Precursor solution preparation

For dielectric deposition, the precursor solution of PMMA was prepared by dissolving PMMA into chlorobenzene (99.8%, Sigma-Aldrich) at a concentration of 20 mg ml^−1^. The solution was subjected to rigorous stirring at 60°C for 8 h. The polymer DPP-DTT, was used in the active channel of the device. To prepare the precursor solution of organic material, DPP-DTT (1-material) was dissolved in chloroform (CHCl_3_, greater than or equal to 99%, Sigma-Aldrich) at a concentration of 6.5 mg ml^−1^. The resulting solution was stirred for 10 h at 60°C to get a homogeneous solution. All chemicals were used as received.

### 4.2. Substrate preparation and deposition

The commercially available highly doped n-type silicon (Si^++^) substrate with pre-grown 400 nm-thick silicon nitride (Si_3_N_4_) acting as the dielectric layer was used as the transistor substrate. Prior to material deposition, the substrate was cleaned by subsequent ultra-sonication in acetone and isopropyl alcohol for 15 min each. The substrate was then blow-dried by a pressurized nitrogen gun. The second dielectric layer (35 nm) was deposited onto the Si_3_N_4_ dielectric by spin-casting the precursor solution of PMMA in an ambient environment at 1000 rpm for 60 s, followed by a post-deposition thermal annealing process for 15 min at 150°C under the nitrogen (N_2_) gas flow rate of 150 l h^−1^. The DPP-DTT solution was then spin coated at 1000 rpm for 60 s in the ambient environment, followed by thermal annealing at 100°C for 20 min under the N_2_ gas flow rate of 150 l h^−1^. The thickness of this layer was measured to be 50 nm by using a surface profilometer.

### 4.3. Contact deposition and characterization

Transistor fabrication was completed by the thermal evaporation of MoO_3_ (5 nm) and Au (100 nm) as metal contacts through a shadow mask under a high vacuum (10^−5^ torr). It resulted in the fabrication of the bottom gate, top contact architect transistor with channel lengths and width of 120 µm and 3 mm, respectively. The electrical measurements of devices were carried out with a Keithly 4200 SCS parameter analyser. The FTIR analysis was performed by Shimadzu ITTracer-100 in ATR mode. All the fabrication and characterization steps were performed in an ambient environment except the annealing process of PMMA and DPP-DTT.

## Data Availability

The data are provided in the electronic supplementary material [55].
